# Orbital Exenteration in Recurrence Cancer: 5 Years Experience

**DOI:** 10.3390/jcm12196180

**Published:** 2023-09-25

**Authors:** Giorgio Barbera, Mattia Todaro, Gianmarco Saponaro, Giulio Gasparini, Alessandro Moro

**Affiliations:** Maxillo Facial Surgery Unit, Fondazione Policlinico Agostino Gemelli IRCCS Hospital, 8 Largo Agostino Gemelli, 00168 Rome, Italy; mattia.todaro@gmail.com (M.T.); gianmarco.saponaro@policlinicogemelli.it (G.S.); giulio.gasparini@policlinicogemelli.it (G.G.); alessandro.moro@policlinicogemelli.it (A.M.)

**Keywords:** exenteration, orbital tumors, temporalis flap, orbital reconstruction

## Abstract

Introduction: The purpose of this study was to assess the overall survival (OS) and disease-free survival (DFS) of patients who underwent orbital exenteration for periorbital, conjunctival, and primary intraorbital carcinomas. Additionally, we assessed the outcomes of anterior retrograde temporalis muscle flap restoration. Methods: For all patients who had orbital exenteration in the previous five years, a non-comparative retrospective assessment of their medical records, histology, and radiographic imaging was carried out. We investigated the relationships between the various qualitative factors using Cramer’s V Kaplan–Meier (KM) analysis. For each of the patient’s categorical factors that were of relevance, estimates of the survival distribution were displayed, and log-rank tests were used to determine whether the survival distributions were equal. Results: This study looks at 19 participants. The sample is made up of 13 men (68%) and 6 women (32%). The degree of relationship (Cramer’s V index) between lymph node metastases (N) and the existence of distant metastases (M) is high, at 64%, and is statistically significant because the *p*-value is 0.0034 < 0.005. Lymph node metastases had a statistically significant impact on overall survival (*p* = 0.04 < 0.05). Thirteen of the nineteen patients tested had no palsy (68%). There was no one presenting a CSF leak. Conclusion: Our findings show how crucial it is to identify any lymph node involvement that orbital neoplasms may have. In patients who have received many treatments, sentinel lymph node biopsy (SLNB) may be used to determine the stage and spread of the cancer. To determine whether additional tumor characteristics may be explored, more expertise in the SLNB field for patients with orbital cancer who have received many treatments may be helpful. To prevent additional scarring and to be comparable to previous techniques for facial nerve lesions, the anterior retrograde approach and the transorbital procedure for temporal muscle flap in-setting are both effective methods.

## 1. Introduction

Orbital exenteration is a surgical technique that involves the removal of the entire eyeball and its surrounding tissues, which include muscles, nerves, fat, and, finally, the eyelids.

A partial exenteration was recorded by Bartische [1] in 1583 as the first account of orbital exenteration surgery. In the early twentieth century, Golovine [2] is credited with publishing the first report on modern complete exenteration.

Orbital exenteration is rarely required in patients with periorbital, conjunctival, and primary intraorbital carcinomas, although it may be necessary in some cases, particularly in patients with numerous recurrences, after multiple eye-sparing treatments, and in patients with high T stages. 

Tumors of the orbit exhibit a variety of etiological, clinical, radiological, and histological variations.

The purpose of this study was to assess overall survival (OS) and disease-free survival (DFS) in patients who had orbital exenteration for periorbital, conjunctival, or primary intraorbital carcinomas. To determine continued therapy before and after the operation, all of the cases were presented at an interdisciplinary tumor meeting. We investigated the prognostic factors in these individuals as well as the outcomes of temporalis muscle flap restoration utilizing an anterograde technique. The goal of this study is to look at the indications, clinical characteristics, outcomes, and survival rates in patients who underwent orbital exenteration for periocular and orbital cancers.

In addition, employing the anterior retrograde technique, we analyzed the results of temporalis muscle flap restoration.

## 2. Methods

All patients who had orbital exenteration in the previous 5 years were subjected to a non-comparative, retrospective evaluation of their medical records, histology, and radiographic imaging.

All of the cases were presented at the interdisciplinary ocular oncology team, which ensures a thorough evaluation of patients through pooling the knowledge of several specialists.

All of the cases are numerous occurrences. Using standard head and neck cancer pathology assessment, free margins of 5 mm have been regarded as ‘close’.

All patients underwent a standardized clinical and neurological assessment prior to surgery. An ophthalmological exam was also performed. Individual advanced diagnostics were then begun based on clinical symptoms, location, and main disease.

We consistently employed the following methods to stage patients:

- A biopsy to confirm the diagnosis;
- MRI of the head and neck with contrast;
- CT of the complete body with contrast.

According to the TNM 8th edition, we included only N0 M0 patients and recurrence T in our study.

Furthermore, individuals who had a follow-up time of less than 3 months or who had missing or incomplete records were excluded from the study.

We defined our exenteration using the Kesting [3] classification.

All cases taken into account in this research were Type I or IIa/b defects according to the Kesting [3] classification. Type I resection is limited to the orbit and includes the removal of the ocular globe, eyelids, retrobulbar soft tissues, and periosteum. Type II is distinguished by the removal of additional orbital wall/rim (IIa) or more (IIb).

The anterior retrograde approach, as reported by Torroni et al. [4], was employed in each patient to raise the temporalis muscle flap. Through the anterior incision utilized to execute the orbital exenteration, the temporalis muscle was widely exposed. Using lateral wall orbitotomy, the harvested flap was rotated and implanted to fill the orbital cavity.

For categorical data, descriptive statistics are displayed as counts and percentages, and for continuous variables, as mean +/− standard deviation. Cramer’s V, an index number used to determine the degree of association between two qualitative statistical features, was utilized to investigate the dependence between the various qualitative variables. For each of the relevant patient features (categorical variables), Kaplan–Meier (KM) estimations of the survival distribution were displayed, and log-rank tests were used to determine if the survival distributions were equal.

All methods used in studies involving human subjects were carried out in conformity with the 1964 Declaration of Helsinki and any later revisions or ethical guidelines that are similar to it.

## 3. Results

Nineteen people are examined in the study. Thirteen males (68%) and six females (32%) make up the sample. The age range taken into account at the time of diagnosis is 30 to 86 years, with an average age of 71 years (SD = 14.64). 

The age box plot Table 1 shows that the key age range is between 67 and 80 years (first and third quartile), which is emphasized.

Thirty percent of the 19 patients had neoadjuvant radiation, which was administered to sixty-eight percent of patients. Only 21% of the 19 patients received chemotherapy, and 50% received neoadjuvant treatment.

**Figure 1:**
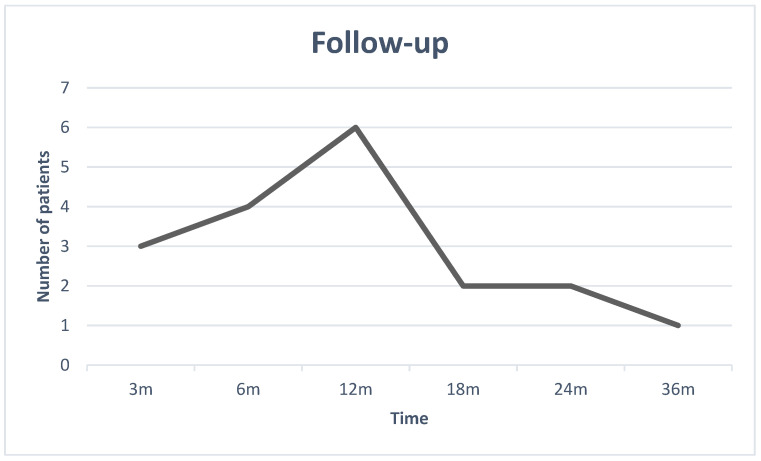
The Follow-up graph.

Through looking at the follow-up graph Figure 1, we can deduce that, out of the 19 patients who were the subject of the study, the mean follow-up length was 1 year, with the shortest follow-up being 3 months and the longest being 3 years. 

Deaths of disease (DODs) are included in the follow-up dates and not displayed individually.

We discovered nine different types of histology (Table 2): five conjunctival carcinomas (26%), one conjunctival melanoma (5%), one ethmoidal sinonasal undifferentiated carcinoma (SNUC) (5%), three eyelid carcinomas, one cutaneous basal cell carcinoma (BCC), one cutaneous squamous cell carcinoma (SCC) (16%), four lacrimal gland adenocarcinomas (21%), two lacrimal gland carcinomas (11%), and one cutaneous melanoma (5%).

According to the TNM AJCC 8th edition, we detected n.2 T2, n.4 T3, and n.13 T4 staging.

According to the T stage, we performed 13 Kesting IIb exenterations, 4 Kesting IIa exenterations, and 2 Kesting I exenterations.

For histology, the Cramer’s V connection index was used; it is high for all three connections investigated with lymph node metastases, distant metastases, and TNM beginning stage.

Although there is a 74% correlation between lymph node metastases and histology, the finding is not statistically significant (*p* = 0.26). Although there is a 66% correlation between distant metastases and histology, this result is not statistically significant (*p* value of 0.56). The correlation between the TNM initial stage and the histology is equivalent to 70%, with a *p*-value of 0.42 indicating that the finding is not statistically significant.

Lymph node metastases (N) were observed in 5 cases out of the 19 patients, (26% of the cases). In three out of the five cases (60%) the node metastases were evident after 3 months, while in the other two cases, after 6 months. 

Metastases (M) were observed in 4 of the 19 patients examined (21%); in 3 of the 4 cases, the patients are the same who had lymph nodes metastases, as well, during the same period. Only in one of the four cases did the patient have negative lymph nodes. 

The degree of connection (Cramer’s V index) between lymph node metastases (N) and the presence of distant metastases (M) is high (64%), with a value considered statistically significant (*p*-value is 0.0034).

Cases with close margins were found in 7 of 19 patients (37%).

Through analyzing the Cramer’s V connection index, we observed a lack of dependence both between the variables of lymph node metastases and close margins and between distant metastases and close margins.

**Figure 2:**
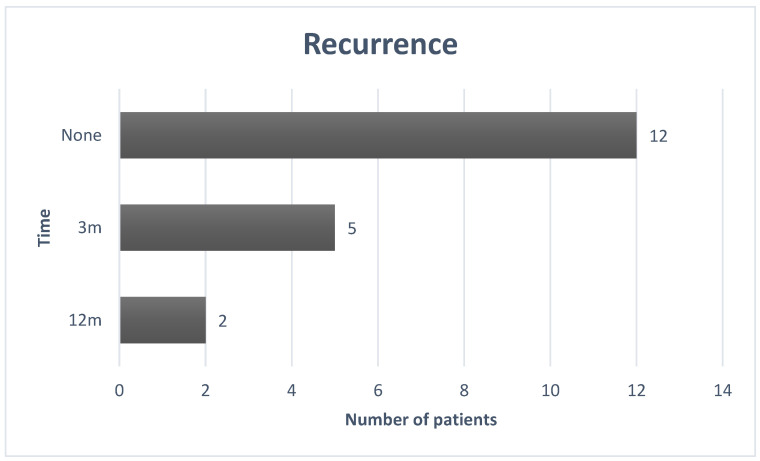
The recurrence graph.

Recurrence was observed in 7 out of 19 patients (37% of the examined cases). Among these patients, 71% had recurrence after 3 months, and the remaining 29%, after one year. As highlighted in the graph (Figure 2), for cases of recurrence, the average is 6 months.

**Figure 3:**
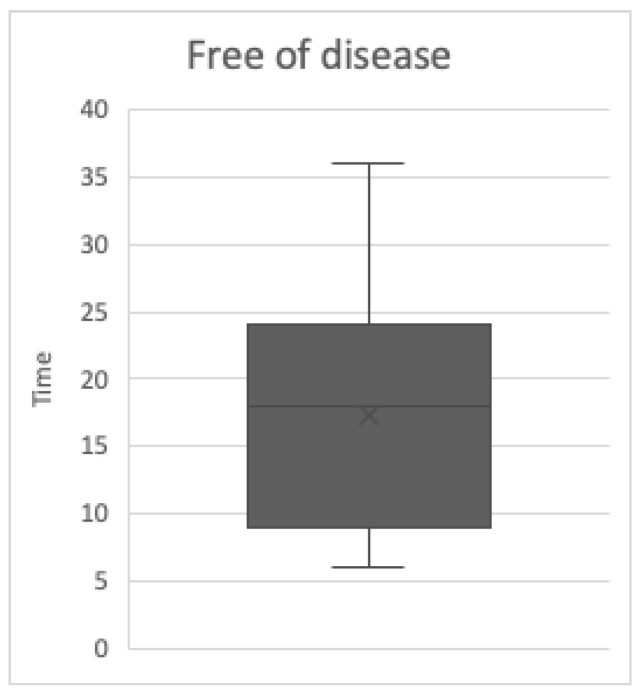
The free of disease (FOD) box plot.

The values of free-of-disease (FOD) patients (9) are stressed by the boxplot (Figure 3): the median is 18 months, the minimum is 6 months, while the maximum is 36 months. The first quartile, which is 12 months, and the third, which is 24 months, are represented by the “box” ends.

### Overall Survival

**Figure 4:**
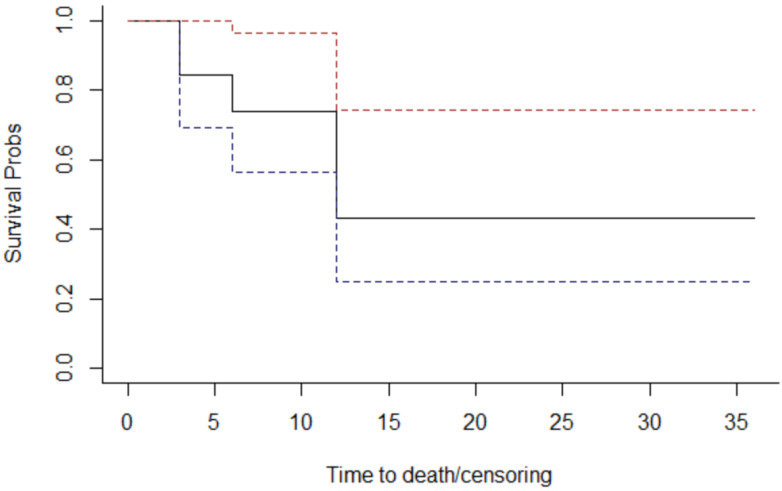
The overall survival graph.

Overall survival is 50% of the total patients, which corresponds to the percentage of deceased patients. The Kaplan–Meier method was used to examine the survival curves of the 19 individuals (Figure 4).

The continuous line indicates the probability of survival as a function of time (expressed in months); discontinuous lines refer to the probability of survival confidence interval.

The probabilities observed at the points where the curve changes have been plotted in the Table 3.

The “Survival” column of the table indicates the probability of survival (the Y-axis of the graph above). For example, we deduce that the probability (cumulative, and not punctual) of survival at the end of the 6th year is 73.7%.

**Figure 5:**
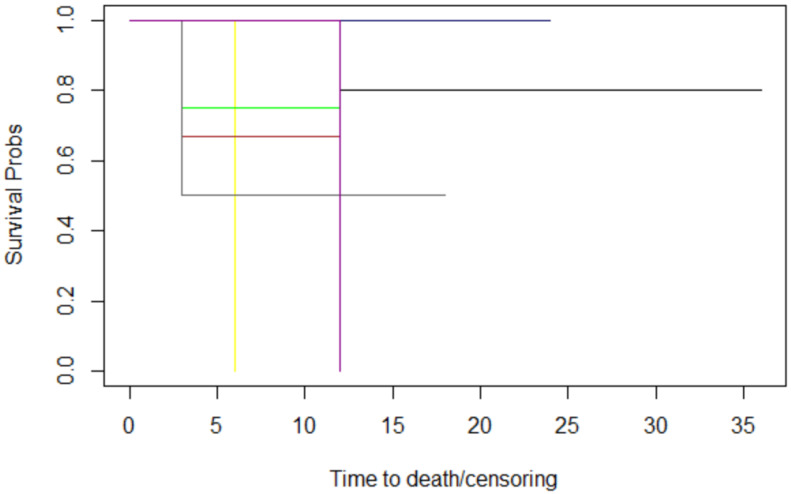
The histology graphs of Kaplan-Meier survival. Histology: conjunctival carcinoma: black; conjunctival melanoma: red; cutaneous BCC: blue; cutaneous SCC: pink; ethmoidal SNUC: yellow; eyelid SCC: brown; lacrimal gland adenocarcinoma: green; lacrimal gland carcinoma: grey; melanoma of the skin: magenta.

**Figure 6:**
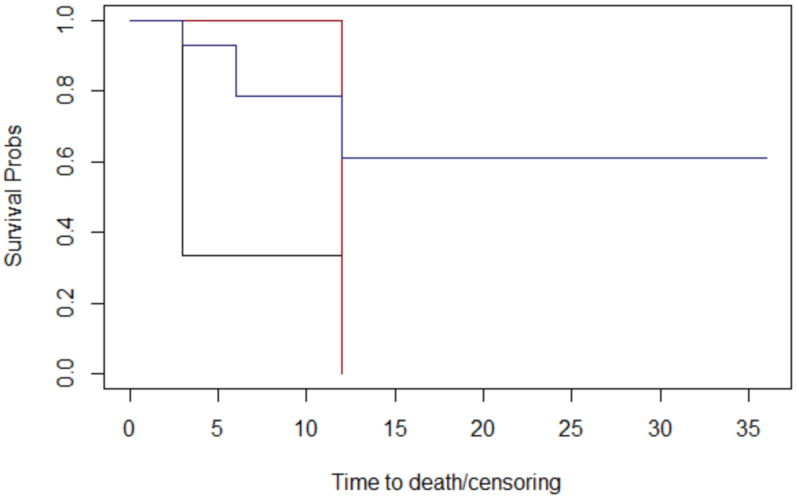
The nodes graphs of Kaplan–Meier survival. lymph nodes metastases: 3 months: black; 6 months: red; No lymph nodes metastases: blue.

**Figure 7:**
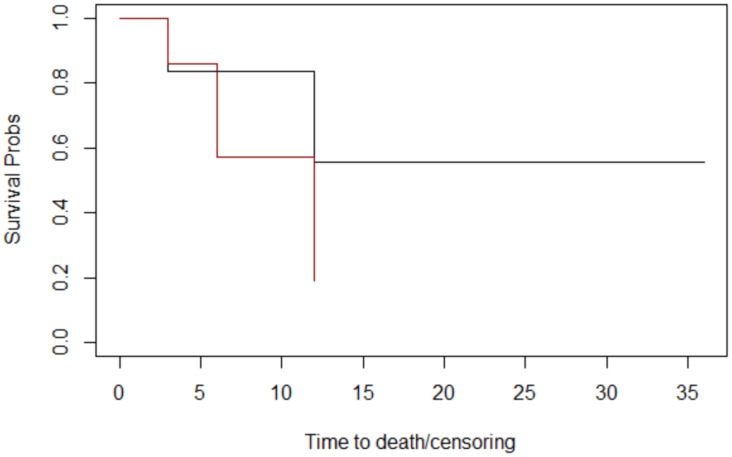
The margins graphs of Kaplan–Meier survival. Margins: close: black; negative: red. The median survival times at follow-up for margins were 18 months for patients whose margins were negative and 9 months for patients whose margins were close.

**Figure 8:**
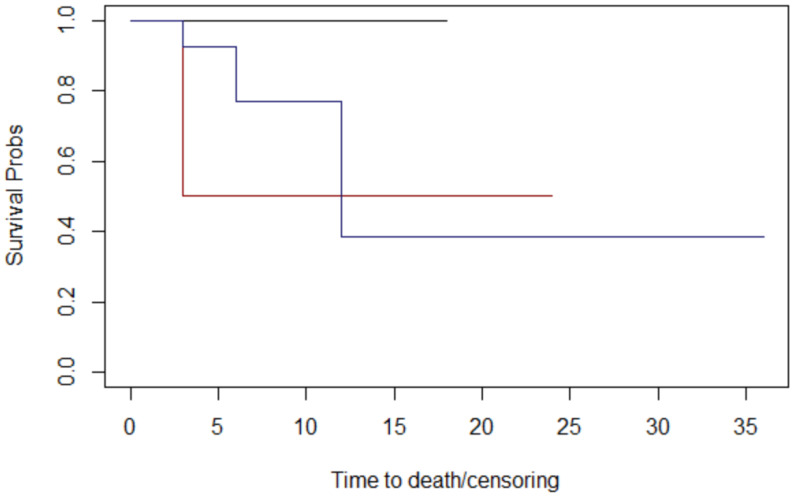
The T stage graphs of Kaplan–Meier survival. T stage: 2: black; 3: red; 4 blue. The median survival times at follow-up for T stage were 12 months for patients with T2, 15 months for patients with T3, and 18 months for patients with T4.

Through assessing histology (Figure 5), lymph node metastases (Figure 6), margins (Figure 7), and T stage (Figure 8), the graphs of Kaplan–Meier survival estimates for the groups under consideration were investigated. 

The log-rank test was then used to compare the variables histology, lymph nodes, margins, and T size. 

The alternative hypothesis is denied in three cases. Therefore histology, margins, and T stage have no effect on survival; nevertheless, the alternative theory is accepted for lymph node metastases.

The computed *p*-values show that there is no difference between histology (*p* = 0.3), margins (*p* = 0.1), and T stage (*p* = 0.4). Lymph node metastases have a significant impact on overall survival (*p* = 0.04 < 0.05) (Table 4).

There were no occurrences of total or partial flap failure.

Thirteen of the nineteen patients studied had no palsy (68%), three had permanent palsy due to surgical injury (16%), two (11%) had earlier palsy (because the nerve was previously invaded by the illness), and one patient had temporary palsy (5%).

No one presented a CSF leak.

## 4. Discussion

Simon [5] reported clear tumor margins after orbital exenteration in 23 cases (68%), while positive margins were obtained in 11 cases (32%), in a sample of 34 patients. Total or extended orbital exenteration was performed on 21 patients (62%), and subtotal exenteration with tissue repair was performed on 13 patients (38%).

Fleming et al. [6] found that of the 25 available data points, a negative margin was established in 23 patients (92%), with an involved margin in 2 patients (8%). The existence of a positive excision margin was the sole variable that demonstrated a significant correlation with a lower DFS (Log-rank 0.0001).

In his research, Baum [7] enrolled 97 patients. According to a univariate study of overall survival, location, neck dissection, lymph node metastases, lymphatic invasion, perineural invasion, resection margins, and immunosuppression were all indicators of a poor prognosis. Resection margins were identified as the sole independent risk factor via multivariate analysis.

In 55 patients, Zhang [8] documented entire orbital exenteration, and in 47 of instances, an anterior exenteration. There was no statistically significant difference in positive surgical margins, recurrences, or mortality in the 57 patients who underwent a lid-sparing operation. Despite obtaining clear surgical margins with orbital exenteration, there were 16 recurrences, with melanomas in particular displaying a high recurrence rate (10 out of 16 recurrences).

According to Aryasit [9], the log-rank test found no statistically significant difference between clear and ambiguous surgical margins in terms of OS (*p* = 0.597).

Seven of the nineteen cases in our analysis (37%) had narrow margins, but the log-rank test and *p*-value did not show that these cases could have an impact on overall survival.

Close margin delineation in orbital and periocular malignancies may need to be revisited, particularly with regard to the periorbita. In fact, the 5 mm and >1 mm criteria may need to be reviewed if the periorbita is likewise devoid of malignancy with a margin less than 5 mm.

Sun [10] came to the conclusion that recurrent tumors had a four-fold higher risk of subsequent recurrence compared to initial tumors, and that a higher T stage was substantially linked with local recurrence. 

Recurrence is noted in 7 out of 19 patients (37%), likely as a result of the high T stages and numerous recurrences in all cases.

Hoffman [11] discovered substantial differences in the time until death with regard to the detection of perineural invasion, lymphovascular invasion, and histopathologic characteristics. However, these differences were not statistically significant.

According to Baum [12], of 190 patients with a malignant tumor, 50 (26.2%) experienced relapses; of these patients, 18 (9.5%) experienced a local recurrence, 22 (11.6%) experienced lymph node metastases, and 10 (5.2%) experienced distant metastases. In total, 67 of their cancer patients passed away (35.2%). All malignant tumors had an OS rate of 77.8%.

Recurrence was noted in 7 out of 19 individuals (37% of the cases that were studied) in our case series. Out of the 19 patients, 5 instances (or 26% of the cases) had lymph node metastases (N).

Four of the nineteen individuals that were assessed (21%) had metastatic disease (M).

Little is known about the reasons to undergo sentinel lymph node biopsy (SLNB) in comparison to tumor characteristics including histology, staging, location, and others (e.g., grading).

Freitag [13] concluded his review through adding that while SLNB is a promising therapy in patients with eyelid and conjunctival cancer, there is currently insufficient evidence to suggest that it improves patient outcomes and survival.

According to our findings, the degree of connection (Cramer’s V index) between lymph nodes and the occurrence of metastases is high, and N metastases have a considerable influence on overall survival. It appears necessary to improve SLNB indications for tumor diagnosis, size, location, and other characteristics.

Ford [14] discovered that for lacrimal gland cancer, orbital exenteration with adjuvant therapy and eye-sparing surgery with adjuvant therapy have identical recurrence results. Better DSS (disease-specific survival) is connected with eye-saving surgery. 

However, 56% of patients in the study had T2 cancer at the time of presentation, and eye-sparing surgery was more common in patients with T1 or T2 tumors at the time of presentation, whereas exenteration was more common in those with T3 or T4 tumors at the time of presentation (*p*-value = 0.006).

Since the FDA (Food and Drug Administration) approved Vismodegib, Sagiv [15] discovered that their practice’s pattern of treating patients with locally advanced periocular BCC has changed. Among patients with comparable clinical characteristics, the prevalence of orbital exenteration was significantly lower, and the rate of treatment with Vismodegib was significantly higher among those treated after the approval of Vismodegib.

Even if employing hedgehog pattern inhibitors in neoadjuvant or adjuvant treatment appears to be the way of the future, the percentile of non-responders is still relatively high.

Gross [16] reported a pathological complete response in 40 patients (51%; 95% confidence interval [CI], 39 to 62) with neoadjuvant therapy with cemiplimab for stage II to IV cutaneous squamous cell carcinoma and a pathological major response in 10 patients (13%; 95% CI, 6 to 22). 

Even if utilizing PD-1 inhibitors in neoadjuvant or adjuvant therapy appears to be the way of the future, the percentile of non-responders is still relatively high.

El-Hadad [17] stated in his report on conjunctival squamous cell carcinoma that the N status is an important prognostic factor for disease-related death, and nodal disease should be evaluated and documented at presentation and during the follow-up surveillance period, especially given the recent availability and approval of immune checkpoint inhibitors such as cemiplimab for the treatment of locally advanced and metastatic SCC.

The interdisciplinary ocular oncology team ensures a full examination of patients through sharing the expertise of different specialists, improving the accuracy of diagnosis and staging, and thus arranging a correct treatment. Because of the complexities of multimodal approaches in cancer therapy, multidisciplinary tumor boards (MDTBs) have recently been formed to create focused, patient-centered treatment plans. However, there are limited data on the applicability of this method in ocular oncology [18].

In terms of reconstruction, the temporalis myofascial flap has generally been regarded as a viable choice. We chose the anterior retrograde technique, which Torroni et al. [4] outlined and which has been shown to be a safe, effective, and viable choice. When compared to free flaps, the temporalis muscle flap significantly reduced morbidity, surgery time, and blood loss. However, if there is no cancer penetration or a small amount of exenteration, it is the best alternative.

According to Kesting et al. [3], locoregional reconstructive procedures like those utilizing the temporalis muscle flap or split-thickness skin grafts have extremely promising outcomes in instances limited to the orbit and/or the orbital walls/rims (Type I and IIa/b). In our experience, the anterior retrograde method is the only one that may be safely employed, produces satisfactory results, and leaves few scars. 

In a prior study, Torroni et al.^4^ noted three cases of temporary facial nerve frontal branch impairment. One patient had a frontal branch impairment that was persistent. Instead, three patients (16%) in the reported series had permanent frontal branch palsy.

The lipofilling of the temporal hollowing brought on by the transfer of the temporalis flap can be used to finish the reconstructive method, according to previous descriptions by Cervelli et al. [19]; they documented 45 patients treated with lipofilling. In each example, there was a distinct soft tissue augmentation of the temporal region. A second procedure was required in 35 individuals, while a third procedure was needed in 1 patient. Lipofilling augmentation is probably a wise choice to regain symmetry when it is possible.

## 5. Conclusions

The typical drawbacks of a retrospective design apply to this study’s retrospective design.

Possibly, T’s varied histology and etiology are its main constraints.

Future studies that are multicentric and comprehensive will undoubtedly be required to assess the behavior of each and every histology. 

Cramer’s V index shows a strong correlation (Cramer’s V index = 64%) between the existence of metastases and the lymph nodes, with a value that is statistically significant (*p*-value = 0.0034).

N metastases have a significant impact on overall survival, according to a log-rank test that was used to examine the impact of each risk factor on survival. 

Based on our findings, it becomes critical to initiate a procedure for sentinel lymph node biopsy (SLNB) and to examine any adjuvant therapy in patients with lymph node micrometastases. Multicenter randomized or case–control studies are required to determine whether tumor sizes, locations, and other features would benefit from SLNB. Furthermore, another research could determine whether detecting and treating nodal micrometastasis improves patient survival.

Data from the literature on radio-guided SNB confirmed that it is practical and safe for conjunctival and eyelid cancers. It may provide predictive information to aid in the selection of the most appropriate treatment management [20].

For both benign and malignant illnesses, whose clinical signs vary greatly from person to person, orbital exenteration continues to be the gold-standard treatment. Exenteration may result from a variety of malignant conditions. Multidisciplinary multimodal therapy may be required in the majority of cases despite a high percentage of R0 resections.

To prevent additional scarring and to be equivalent to previous techniques for facial nerve lesions, the anterior retrograde route and the transorbital method for temporal muscle flap insetting are both effective methods.

The examination of the frontal branch paralysis data shows that, on average, patients do not report facial paralysis, with the exception of 16%.

## Figures and Tables

**Table 1:** The age box plot.

VAR	MIN	1° Quart.	Median	Media	3° Quart.	MAX	Sd.
Age	30.00	67.50	74.00	70.84	80.50	86.00	14.64

**Table 2:** Types of histology.

Histopathological Diagnoses	Patients (*n* = 19)
Conjunctival carcinoma	5
Lacrimal gland adenocarcinoma	4
Eyelid SCC	3
Conjunctival melanoma	1
Cutaneous BCC	1
Cutaneous SCC	1
Ethmoidal SNUC	1
Lacrimal gland carcinoma	2
Melanoma of the skin	1

**Table 3:** The “Survival” Table.

Time	N. Risk	N. Event	Survival	Std. Error	Lower 99% CI	Upper 95% CI
3	19	3	0.842	0.0837	0.693	1.000
6	16	2	0.737	0.1010	0.563	0.964
12	12	5	0.430	0.1203	0.248	0.744

**Table 4:** The log-rank test calculated for variables.

Variable	N°obs. Per Group	Variance	Log-Rank Test	*p*-Value
**Histology**			9.90	0.30
conjunctival carcinoma	5	3.6885		
conjunctival melanoma	1	0.3302		
cutaneous BCC	1	1.0082		
cutaneous SCC	1	2.1220		
ethmoidal SNUC	1	2.1220		
eyelid SCC	3	2.0808		
lacrimal gland adenoK	4	0.0219		
lacrimal gland carcinoma	2	0.0337		
melanoma of the skin	1	0.1860		
**Lymph nodes metastases**			6.50	0.04
3 months	3	5.571		
6 months	2	0.402		
No lymph nodes metastases	14	4.782		
**Margins**			2.2	0.10
Close margin	7	2.17		
No	12	2.17		
**T stage**			1.70	0.40
T = 2	2	1.382		
T = 3	4	0.553		
T = 4	13	0.057		

Log-rank test calculated for variables: histology, lymph nodes metastases, margins and T stage.

## Data Availability

Not applicable.
